# Glycolytic metabolism is essential for CCR7 oligomerization and dendritic cell migration

**DOI:** 10.1038/s41467-018-04804-6

**Published:** 2018-06-25

**Authors:** Hannah Guak, Sara Al Habyan, Eric H. Ma, Haya Aldossary, Maia Al-Masri, So Yoon Won, Thomas Ying, Elizabeth D. Fixman, Russell G. Jones, Luke M. McCaffrey, Connie. M. Krawczyk

**Affiliations:** 1 Goodman Cancer Research Centre, Department of Physiology, McGill University, Montreal, QC H3G 1Y6 Canada; 2 Goodman Cancer Research Centre, Department of Oncology, McGill University, Montreal, QC H3G 1Y6 Canada; 3 Meakins-Christie Laboratories, Research Institute of McGill University Health Center, Department of Medicine, McGill University, Montreal, H4A 3J1 QC Canada; 4 Goodman Cancer Research Centre, Department of Microbiology and Immunology, McGill University, Montreal, QC H3G 1Y6 Canada

## Abstract

Dendritic cells (DCs) are first responders of the innate immune system that integrate signals from external stimuli to direct context-specific immune responses. Current models suggest that an active switch from mitochondrial metabolism to glycolysis accompanies DC activation to support the anabolic requirements of DC function. We show that early glycolytic activation is a common program for both strong and weak stimuli, but that weakly activated DCs lack long-term HIF-1α-dependent glycolytic reprogramming and retain mitochondrial oxidative metabolism. Early induction of glycolysis is associated with activation of AKT, TBK, and mTOR, and sustained activation of these pathways is associated with long-term glycolytic reprogramming. We show that inhibition of glycolysis impaired maintenance of elongated cell shape, DC motility, CCR7 oligomerization, and DC migration to draining lymph nodes. Together, our results indicate that early induction of glycolysis occurs independent of pro-inflammatory phenotype, and that glycolysis supports DC migratory ability regardless of mitochondrial bioenergetics.

## Abstract (web summary)

The activation of dendritic cells (DC) is associated with a metabolic switch from oxidative to glycolytic metabolism. Here, the authors show that both strong and weak stimuli cause an immediate increase in glycolysis, but only strong stimuli induce long-term glycolytic reprogramming.

## Introduction

Dendritic cells (DCs) are among the first responding immune cells to any infection, injury, or threat. DCs express a wide variety of pattern recognition receptors (PRRs), which are germline-encoded receptors that recognize conserved moieties such as non-self microbe/pathogen-associated molecular patterns and danger-associated molecular patterns, often released during cell death or injury. Membrane-bound PRRs include Toll-like receptors (TLRs) and C-type lectin receptors (CLRs). DCs adopt different activation phenotypes depending on the combination of receptors engaged and the context in which activation occurs; this process is known as differential activation. Differential activation enables DCs to transmit context-specific information to other cells of the immune system and consequently direct the nature of inflammatory responses.

Regardless of their activation phenotype, DCs migrate from peripheral tissues to the draining lymph node (LN) where they interact with other cells of the immune system. For example, the activation phenotype of DCs directly determines T helper (Th) cell differentiation. DCs that are stimulated with TLR ligands such as lipopolysaccharide (LPS) stimulate a pro-inflammatory phenotype characterized by production of cytokines such as interleukin (IL)-12, IL-6, tumor necrosis factor (TNF)-α, and moderate amounts of IL-10, and promote the differentiation of naive Th cells into interferon (IFN)-γ-producing Th1 cells^1^. DCs stimulated by the yeast component zymosan (Zym), which engages TLR2, TLR6, and the CLR Dectin-1, produce less IL-12 and more IL-10 relative to LPS-stimulated DCs, and promote the induction of Th17 cells^2, 3^. DCs that encounter the allergen house dust mite (HDM) engage TLR2, TLR4, and Dectin-2, and produce very little pro-inflammatory cytokines, but are able to promote the activation and differentiation of Th2 and Th17 cells^4, 5^.

In recent years, cellular metabolism has become recognized as an important determinant of immune cell inflammatory phenotype and function^6–12^. Glycolysis and oxidative phosphorylation (OXPHOS) are the main bioenergetic catabolic pathways, taking place in the cytosol and mitochondria, respectively. Glucose-derived pyruvate can either be converted to lactic acid and expelled from the cell, or completely oxidized in the mitochondria via the tricarboxylic acid (TCA) cycle. The TCA cycle fuels OXPHOS by providing reducing agents to drive the electron transport chain, consuming oxygen as the final electron acceptor. Although OXPHOS supports a higher yield of ATP per molecule of glucose, induction of glycolytic metabolism despite the presence of oxygen (i.e., the Warburg effect) is observed in many cell types^13^. We and others have shown that TLR-mediated DC activation results in a striking Warburg-like metabolic shift to glycolysis, which is necessary to support their pro-inflammatory phenotype^7, 14, 15^. The immediate increase in glycolytic activity in TLR-activated DCs was found to be required for de novo fatty acid synthesis to expand the endoplasmic reticulum and Golgi to support the increased production and secretion of immune mediators^14^. In long-term activation of inducible nitric oxide synthase (iNOS)-expressing DCs, TLR-induced nitric oxide (NO) production was shown to block electron transport, resulting in the cessation of OXPHOS^15^. Thus, in NO-producing cells, the increase in glycolysis was found to be only necessary in the absence of OXPHOS^15^. In DCs lacking iNOS, a long-term shift to glycolytic metabolism also occurs, although this is thought to be driven by autocrine type I IFN signaling through hypoxia-inducible factor-1α (HIF-1α)^16^.

The importance of metabolic programming for the function of DCs has largely been shown for highly pro-inflammatory DCs; however, activated DCs can take on distinct activation phenotypes. We therefore examined the metabolic profiles of DCs activated by a more diverse range of stimuli, including both weak and strong activators of DCs, to determine the impact of glycolytic metabolism on DC function. We found that regardless of activation stimulus, DCs increase their glycolytic activity following stimulation. DCs activated with strong stimuli such as LPS or Zym display increased glycolysis with a corresponding decrease in OXPHOS. However, we found that HDM-activated DCs, which display a weak pro-inflammatory phenotype, also rapidly increased glycolysis early following stimulation, but lacked long-term metabolic reprogramming. TBK, AKT, and mTOR signaling pathways, which are known to regulate glycolysis in DCs, were activated in DCs with either a weak or strong pro-inflammatory phenotype. However, long-term activation of these pathways and increased HIF-1α activation occurred only in DCs with a highly pro-inflammatory phenotype. We demonstrate that glycolysis, and not mitochondrial metabolism, is important for DC motility and migration, as inhibition of glycolysis impaired the maintenance of elongated cell shape, DC motility, and DC migration to draining LNs in vivo. Importantly, glucose metabolism is also necessary for CCR7 oligomerization, which is essential for efficient DC migration. These findings reveal that glycolysis induction occurs independent of acquisition of a pro-inflammatory phenotype and supports the migratory capacity of DCs.

## Results

### Differentially activated DCs display distinct bioenergetic profiles

**Fig. 1:**
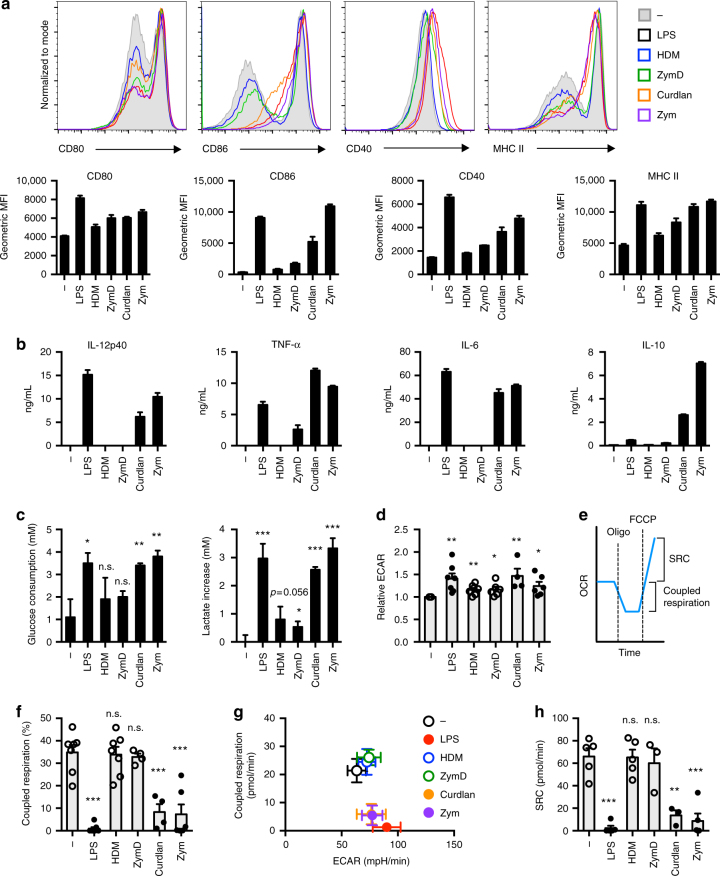
Bioenergetic profiles of differentially activated DCs. **a**, **b** Activation marker expression (CD80, CD86, CD40, and MHC II) and cytokine production (IL-12p40, TNF-α, IL-6, and IL-10) were measured following 18 h activation with indicated activators. **c** Glucose consumption (left) and lactate increase compared to the unstimulated condition (right) were measured 18 h following stimulation. **d**, **f**–**h** ECAR and OCR were measured using a Seahorse Bioanalyzer following an 18 h stimulation. **e** Coupled respiration is represented by the decrease in OCR from baseline after oligomycin treatment, and is therefore the OCR attributed to ATP production. Spare respiratory capacity (SRC) is represented by the increase in OCR from baseline after FCCP treatment. **f** Coupled respiration as a percentage of total respiration. **g** Mean coupled respiration versus mean ECAR. **h** SRC. Data shown in **a**–**c** are from one experiment representative of at least three independent experiments (mean and s.d. of triplicates (**a**, **c**) or duplicates (**b**)). Data shown in **d**, **f**–**h** are from three to seven experiments (depending on condition) (mean and s.e.m.). LPS: lipopolysaccharide, HDM: house dust mite, ZymD: zymosan depleted of TLR ligands, Zym: zymosan, ECAR: extracellular acidification rate, OCR: oxygen consumption rate. Data were analyzed using *t*-test, comparing each condition relative to the control. ***p < 0.001, **p < 0.01, *p < 0.05

Stimulation of DCs with strong TLR agonists such as LPS promotes metabolic reprogramming to glycolysis with concomitant loss of ATP-coupled mitochondrial respiration^7, 14, 17^. To assess whether this is a universal effect of PRR stimulation, we examined the bioenergetic profile of DCs stimulated with a range of activators that engage different PRRs (Table 1), resulting in different inflammatory phenotypes. We measured surface expression of the co-stimulatory molecules CD80, CD86, CD40, and major histocompatibility complex (MHC) II (Fig. 1a, Supplementary Fig. 1a), as well as the production of cytokines IL-12p40, TNF-α, IL-6, and IL-10 (Fig. 1b, Supplementary Fig. 1b) following stimulation with these activators. “Strong” activators (i.e., LPS, Zym, and curdlan) induced much greater surface marker expression and cytokine secretion compared to “weak” activators (i.e., HDM and Zym depleted of its TLR ligands (ZymD)).

**Table 1:** DC activators

Activator	PRRs engaged	Concentration
Lipopolysaccharide (LPS)	TLR4	10 or 100 ng/mL
House dust mite (HDM)	TLR2/4, Dectin-2	50 μg/mL
Curdlan	Dectin-1	25 or 50 μg/mL
Zymosan	TLR2/6, Dectin-1	5 or 10 μg/mL
Zymosan-depleted (ZymD)	Dectin-1	10 or 25 μg/mL

To assess metabolic reprogramming induced by these activators, we examined glucose consumption and lactate production in DCs 18 h following stimulation with either strong or weak DC activators. All DC activators stimulated increased glucose consumption and lactate production relative to resting DCs, with strong DC activators generally displaying higher levels of glucose metabolism than weaker activators (Fig. 1c). We next used Seahorse profiling, measuring the extracellular acidification rate (ECAR, a measure of glycolysis) and oxygen consumption rate (OCR, Fig. 1e), to determine the bioenergetic profiles of differentially activated DCs. Consistent with previously published reports^7, 14, 17^, resting DCs engaged both OXPHOS and glycolysis, while LPS-treated DCs no longer displayed ATP-coupled mitochondrial respiration and exclusively used glycolysis for ATP production (Fig. 1d, f, g). Similar to LPS, stimulation with the highly pro-inflammatory activators curdlan and Zym increased ECAR and decreased coupled respiration (Fig. 1d, f). Interestingly, weak DCs agonists (HDM and ZymD) induced slight but consistent increases in ECAR while retaining coupled respiration (Fig. 1d, f).

The difference in maximal mitochondrial respiratory capacity, measured after fluoro-carbonyl cyanide phenylhydrazone (FCCP) treatment, and basal respiration is known as the spare respiratory capacity (SRC) (Fig. 1e, h). DCs stimulated with strong activators (LPS, curdlan, and Zym) most often displayed negligible SRC, although in some experiments DCs stimulated with curdlan and Zym retained SRC, while the SRC of weakly activated DCs was consistently similar to resting DCs (Fig. 1h). These data indicate that differential DC activators can induce intermediate bioenergetic phenotypes in DCs, resulting in the stimulation of glycolysis while retaining varying degrees of OXPHOS and mitochondrial respiratory capacity.

### Early glycolytic flux is a hallmark of both weak and strong DC activators

**Fig. 2:**
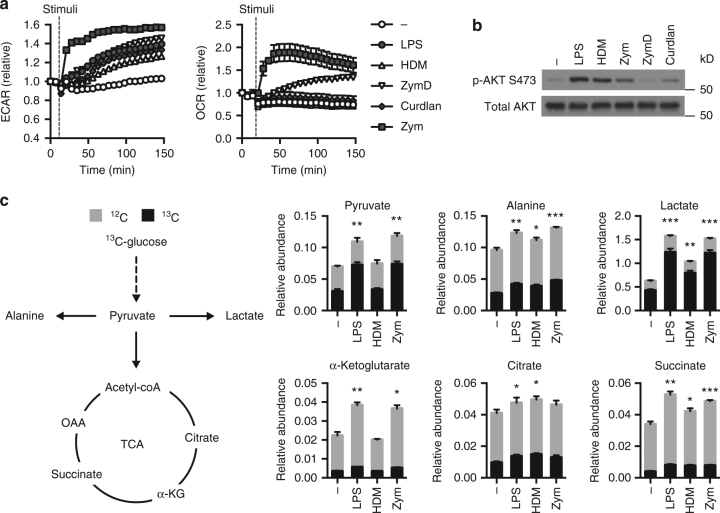
Weakly activated DCs undergo early glycolytic flux. **a** Indicated stimuli were injected onto DCs using the Seahorse Bioanalyzer and ECAR (left) and OCR (right) were measured in real-time. **b** Immunoblot for total AKT and AKT phosphorylated at Ser473 in DCs following 1 h of stimulation. **c** The relative abundance of various metabolites determined by GC-MS after activation for 2 h and pulse with ^13^C-glucose. Data shown are one experiment representative of (**a**) more than three experiments, (**b)** three experiments, and (**c**) two experiments (mean and s.e.m. of 9 (unstimulated) or 10 (stimulated) replicates (**a**) or triplicates (**c**)). Statistical analysis shown in **c** is for the comparison of the ^13^C-labeled fraction between each condition and the control condition using unpaired *t*-tests. ***p < 0.001, **p < 0.01, *p < 0.05

Given that both strongly and weakly activated DCs increase glycolysis, regardless of their pro-inflammatory nature, we monitored the bioenergetic states (ECAR and OCR) of differentially activated DCs in real-time following PRR stimulation. All differentially activated DCs, including those weakly activated by HDM or ZymD, displayed increased ECAR immediately after stimulation (Fig. 2a, left). Short-term activation did not change the OCR of differentially activated DCs, except those stimulated with Zym and ZymD (Fig. 2a, right). The increase in oxygen consumption observed with these stimuli was determined to be largely non-mitochondrial (Supplementary Fig. 2), which is consistent with literature demonstrating that Zym stimulates NADPH oxidase activity and the consequent production of reactive oxygen species^3, 18^. The serine/threonine protein kinase AKT is known to be required for glycolytic activity in TLR-activated DCs^14^. Analysis of phospho-AKT (Ser473) levels following stimulation for 1 h revealed increased, albeit variable, AKT activation in differentially activated DCs regardless of inflammatory stimulus (Fig. 2b). Thus, the downregulation of OXPHOS observed in strongly stimulated DCs is a long-term adaptation to PRR stimulation, while AKT activation and increased glycolysis is an early event following activation and is independent of a pro-inflammatory phenotype.

To further characterize glucose metabolism following DC activation, we investigated the fate of ^13^C-labeled glucose in activated DCs using stable isotope tracer analysis (Fig. 2c)^19^. DCs were activated with LPS, HDM, or Zym for 2 h and pulsed with U-[^13^C]-glucose for an additional 2 h (Fig. 2c). Zym- and LPS-stimulated DCs displayed hallmark profiles of Warburg metabolism, characterized by increased conversion of ^13^C-glucose to ^13^C-pyruvate and ^13^C-lactate (Fig. 2c). Consistent with their bioenergetic profile (Fig. 1), HDM-stimulated DCs displayed increased ^13^C-lactate production from ^13^C-glucose, although at lower levels than observed in LPS- or Zym-stimulated DCs (Fig. 2c). Moderate but consistent increases in ^13^C-glucose-derived TCA cycle intermediates were observed in activated DCs (Fig. 2c), suggesting evidence of glucose conversion to acetyl-CoA. Of note was an increased abundance of unlabeled α-ketoglutarate and succinate in strongly activated DCs (Fig. 2c), suggesting an alternative carbon source is contributing to the increased production of these metabolites. DCs also converted a significant amount (~30%) of ^13^C-glucose to alanine, indicating that glucose-derived pyruvate has several metabolic fates—lactate, alanine, and acetyl-CoA—which are all produced independent of pro-inflammatory phenotype.

### Loss of coupled respiration directly correlates with iNOS expression and NO production

**Fig. 3:**
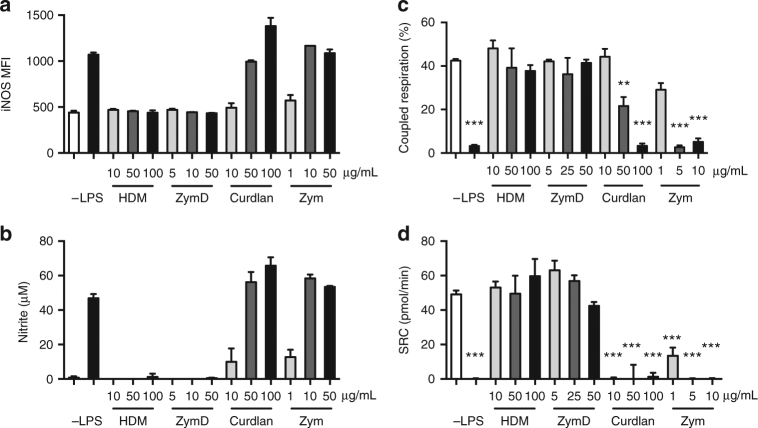
iNOS expression and NO production is correlated with loss of coupled respiration. DCs were stimulated for 18 h by activators at the indicated doses (μg/mL). **a** Geometric MFI of iNOS, **b** nitrite (NO_2_^−^) production, **c** coupled respiration, and **d** SRC were measured as described in Methods and in Fig. 1. Data shown are from one experiment representative of three experiments (mean and s.d. of duplicates (**a**, **b**) and s.e.m. of four to six replicates (**c**, **d**)). Data were analyzed using a one-way ANOVA. ***p < 0.001, **p < 0.01

Increased glycolytic metabolism in LPS-stimulated DCs has been reported to be required for energy production due to the inhibition of mitochondrial respiration by NO^15^. To determine whether loss of mitochondrial metabolism also correlates with NO production in differentially activated DCs, iNOS expression and NO production were measured in DCs activated by various stimuli. iNOS expression increased in a dose-dependent manner for curdlan and Zym (levels plateaued after 10 μg/mL for Zym) (Fig. 3a), with corresponding increases in nitrite levels (Fig. 3b). In these strongly activated DCs, the increase in iNOS expression and NO production correlated with the loss of coupled respiration (Fig. 3c). Inhibition of mitochondrial oxidative metabolism was further reflected by the little to no SRC remaining in the strongly activated DCs (Fig. 3d). No appreciable levels of iNOS or NO were induced by HDM or ZymD stimulation of DCs (Fig. 3a, b), and both coupled respiration and SRC were maintained in these activated DCs (Fig. 3c, d). Together, these data show that iNOS expression and NO production correlates with the loss of coupled respiration in differentially stimulated DCs and that an early increase in glycolysis can occur regardless of whether iNOS is later induced.

### Weakly inflammatory DCs lack long-term glycolytic reprogramming

**Fig. 4:**
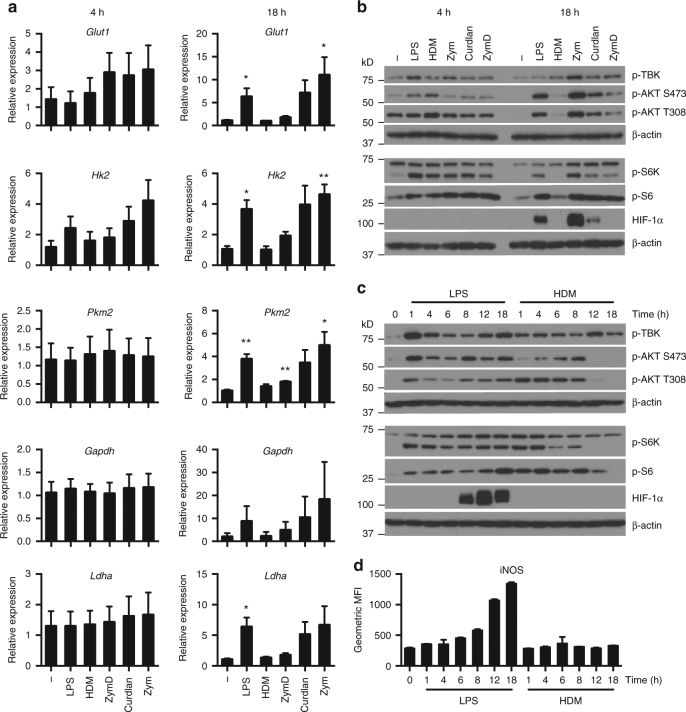
Weakly activated DCs lack long-term metabolic reprogramming. **a** Gene expression of glucose transporter *Glut1* and glycolytic enzymes *Hk2, Pkm2, Gapdh*, and *Ldha* by DCs were measured after differential activation for 4 h (left) and 18 h (right). **b**, **c** Immunoblot for p-AKT (Ser473), p-AKT (Thr308), p-TBK (Ser172), p-S6K, p-S6, HIF-1α, and β-actin following (**b**) differential activation for 4 and 18 h, and (**c**) activation by LPS or HDM over a time course from 1 to 18 h. **d** Geometric MFI of iNOS in DCs activated by LPS or HDM over a time course from 1 to 18 h. Data shown in **a** are fold changes from three experiments, **b**, **c** one experiment representative of three experiments, and **d** one experiment representative of two experiments (mean and s.e.m. (**a**) and s.d. (**d**) of triplicates). Data were analyzed using *t*-test, comparing each condition relative to the control. **p < 0.01, *p < 0.05

Metabolic reprogramming in immune cells is reinforced by changes in expression of key metabolic pathway genes that underlie changes at the biochemical level. To assess the impact of different TLR agonists on DC metabolic reprogramming, we examined the kinetics of gene expression of key enzymes in glycolysis following activation. During early stages of DC activation (4 h post stimulation), minor changes in the expression of *Glut1* and *Hk2* were observed, but expression of distal components of glycolysis (*Pkm2*, *Gapdh*, and *Ldha*) were unchanged (Fig. 4a, left). However, at late time points (18 h post stimulation), DCs activated with pro-inflammatory stimuli (LPS, curdlan, and Zym) showed increased expression of glycolytic genes, whereas DCs stimulated with “weak” activators HDM and ZymD showed little or no change in the expression of these enzymes relative to resting DCs (Fig. 4a, right). These results suggest that increased early glycolysis induced by stimulation occurs independent of large-scale changes in metabolic gene expression. Metabolic reprogramming at the level of transcription occurs following long-term DC activation in strongly pro-inflammatory DCs, but not in weakly activated DCs.

The TBK-IKKε signaling pathways have been identified as key kinases important for early induction of glycolysis in DCs^14^. We found that early activation of TBK, AKT, and mTORC1 occurred regardless of the activation phenotype (Fig. 4b). However long-term activation of both mTORC1 (measured by phospho-S6K and phospho-S6 levels) and mTORC2 (measured by phospho-AKT Ser473 levels) was associated only with the presence of a pro-inflammatory phenotype (Fig. 4b). Analysis of the time course revealed that AKT and mTORC1 activity was progressively lost in HDM-stimulated DCs around 8–12 h following activation (Fig. 4c). Early glycolysis induction by HDM, like LPS^14^, could be prevented by AKT inhibition (Supplementary Fig. 3). Interestingly, TBK phosphorylation levels were maintained in HDM-stimulated cells (Fig. 4c). Stable HIF-1α protein expression was observed in LPS-stimulated DCs between 8–18 h post activation, but absent in HDM-stimulated DCs at all time points (Fig. 4c). Consistent with the role of HIF-1α in glycolytic reprogramming, the absence of detectable HIF-1α protein levels in DCs stimulated with weak agonists correlated with the lack of significant long-term induction of HIF-1α-dependent glycolytic gene expression (*Hk2* and *Ldha*)^20^ in these cells (Fig. 4a). Induction of iNOS was also absent in HDM-stimulated DCs (Fig. 4d). These data are consistent with evidence linking mTORC1 to long-term commitment to glycolysis by induction of iNOS and HIF-1α^21^.

### Induction of glycolysis is required for DC motility and migration

**Fig. 5:**
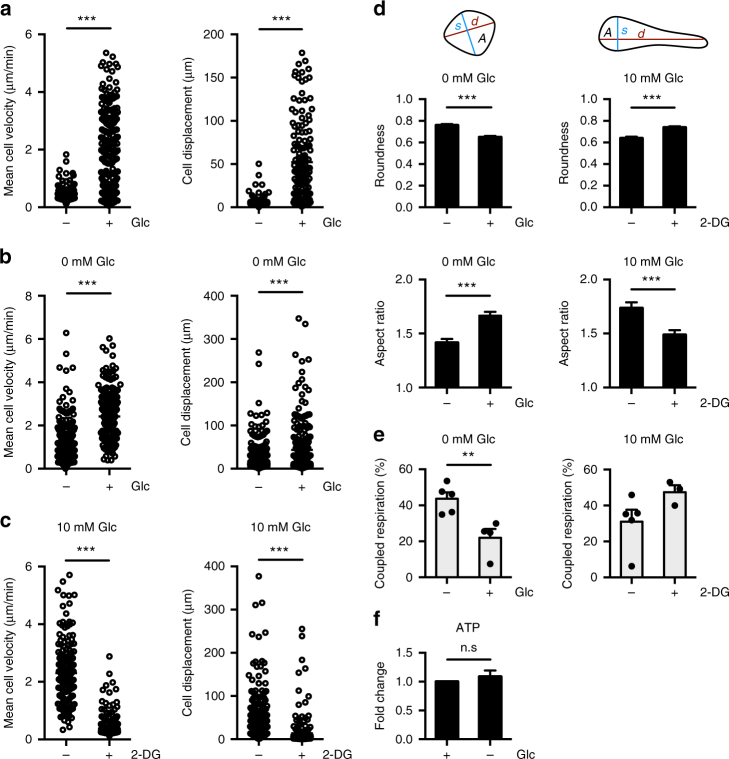
Glycolysis is required for DC motility and morphology. Images of DCs were captured over time using confocal microscopy. **a** Mean cell velocity (left) and cell displacement (right) of DCs in the presence or absence of 10 mM glucose after 4 h. **b** Mean cell velocity (left) and cell displacement (right) of DCs cultured in the absence of glucose following the addition of media with or without 10 mM glucose. **c** Mean cell velocity (left) and cell displacement (right) of DCs cultured in the presence of glucose after the addition of media with or without 10 mM 2-deoxyglucose (2-DG). **d** Roundness (4 × *A*/(*π* × *d*^2^); top left) and aspect ratio (*d*/*s*; bottom left) of DCs cultured without glucose after the addition of media with or without 10 mM glucose, and roundness (top right) and aspect ratio (bottom right) of DCs cultured in the presence of glucose after the addition of media with or without 10 mM 2-DG. **e** Coupled respiration as a percentage of total respiration of DCs cultured without glucose and following the addition of glucose (left), and of DCs cultured in the presence of glucose and following the addition of 2-DG (right). **f** Fold change of ATP levels in DCs cultured with or without glucose relative to glucose condition. Data in **a** represent 164–210 cells. Data in **b**–**d** represent 179–268 cells from one experiment representative of two experiments. Data in **e** are three to five experiments (depending on condition) (mean and s.e.m.). Data in **f** are fold changes of three experiments (mean and s.e.m.). Data were analyzed using unpaired *t*-tests. ***p < 0.001, **p < 0.01, *p < 0.05

Our results indicate that early glycolytic flux is necessary for an aspect of DC function beyond sustaining a pro-inflammatory phenotype. Activated DCs, regardless of activation phenotype, must migrate to the draining LN to stimulate immune responses. Therefore, we examined the impact of glucose availability on DC migration. Using live imaging via confocal microscopy, we determined the cell velocity and displacement (distance traveled) in the presence or absence of glucose in culture medium. Under glucose-limiting conditions, DCs exhibited dramatic reductions in motility, characterized by both reduced velocity and shorter overall distance traveled (Fig. 5a, Supplementary Movie 1). To ensure that reduced DC motility was not due to reduced cell viability, we cultured DCs in glucose-free medium and measured their motility upon the addition of glucose (10 mM). DCs immediately increased both velocity and displacement upon addition of glucose, displaying motility equal to that of DCs initially cultured in the presence of glucose (Fig. 5b, c, Supplementary Movie 2). Adding the glycolytic inhibitor 2-deoxyglucose (2-DG) significantly decreased DC velocity and displacement (Fig. 5c), implicating glycolysis in this process. Culturing DCs without glucose or with 2-DG also impacted DC morphology, stimulating the cells to become more round with retraction of dendrites (Fig. 5d, Supplementary Fig. 4). When glycolytic activity was reduced, either by glucose withdrawal or culture with 2-DG, DCs displayed metabolic compensation to mitochondrial OXPHOS (Fig. 5e), allowing DCs to maintain cellular ATP levels (Fig. 5f). These data demonstrate that DCs are capable of metabolic plasticity (shifting between glycolysis and OXPHOS) to maintain ATP production. However, increased OXPHOS is not sufficient to restore DC motility, and thus glycolysis is essential for DC motility. These data suggest that glycolysis is necessary for cytoskeletal changes that support cell shape and motility.

**Fig. 6:**
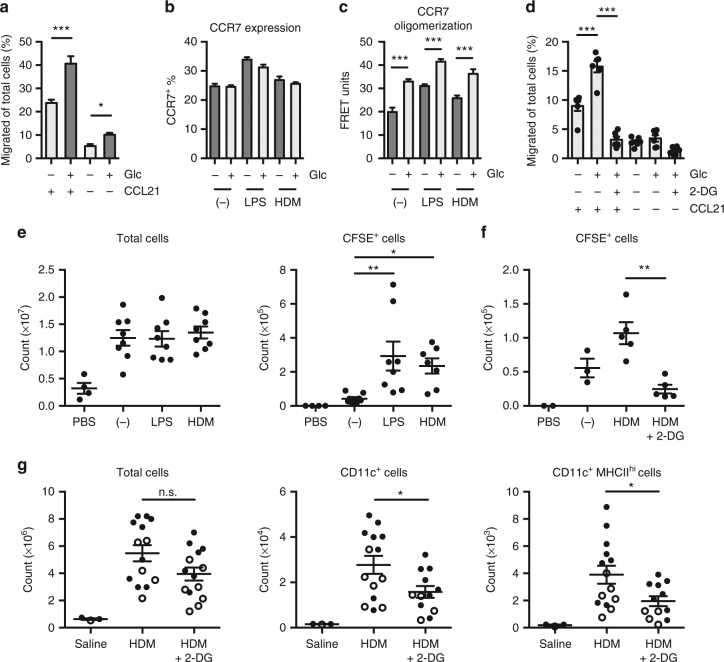
Inhibition of glycolysis impairs CCR7 oligomerization and reduces DC migration to the draining lymph node. **a** DCs incubated with or without 10 mM glucose for 4 h migrate toward CCL21 for 2 h. **b** Total CCR7 expression levels were measured and **c** CCR7 oligomerization was determined by FRET of DCs left unstimulated or stimulated with LPS or HDM with or without 10 mM glucose for 6 h. **d** DCs isolated from the spleen were cultured with or without 10 mM glucose or 2-DG for 6 h, then migrate toward CCL21 for 2 h. **e** CFSE-labeled DCs stimulated were injected subcutaneously into the footpads of mice and the popliteal LNs were harvested after 45 h. Total cell number (left) and CFSE^+^ cells (right) from dLNs were determined. **f** CFSE-labeled DCs were stimulated with HDM with or without 10 mM 2-DG prior to footpad injection and LN harvest as in **e**. **g** Cell numbers from mediastinal LNs of mice treated i.n. with 40 μg HDM or saline, and i.p. with 2-DG or saline. Data in **a**–**c** are from one experiment representative of three experiments (mean and s.d. of triplicates). Data in **d** and **e** are from two experiments (mean and s.e.m. of 6 mice (**d**), 4 mice for PBS control and 8 mice for other conditions (**e**)). Data shown in **f** are from one experiment representative of three experiments (mean and s.e.m. of 2 mice (PBS), 3 mice (unstimulated), or 4–5 mice (other conditions)). Data in **g** are from two experiments, with closed and open circles representing mice from different experiments (mean and s.e.m. of 3 mice for saline control and 14 mice for other conditions). Data were analyzed using one-way ANOVA. ***p < 0.001, **p < 0.01, *p < 0.05

In vivo, upregulation of CCR7, the receptor for the chemokines CCL21 and CCL19, in response to activating stimuli is necessary for DC migration to the draining LN. We found that in vitro, glucose was essential for DC migration toward CCL21 (Fig. 6a). Glucose limitation had little effect on overall CCR7 expression on either resting or activated DC populations, however decreased CCR7 expression on CCR7^+^ DCs was observed (Fig. 6b, Supplementary Fig. 5). Oligomerization of CCR7 monomers enables efficient DC migration moreso than expression levels, and is necessary to guide DCs to the draining LN^22^. By fluorescence resonance energy transfer (FRET), we determined that CCR7 oligomerization on DCs was significantly impaired under conditions of reduced glucose availability, both in the absence and presence of activating stimuli (Fig. 6c). Finally, we examined DC migration toward CCL21 by splenic DCs, and found that, like bone marrow-derived DCs (BMDCs), splenic DC migration toward CCL21 was reduced upon glucose limitation or when glycolysis was blocked by 2-DG (Fig. 6d).

To determine the impact of glycolysis on DC migration in vivo, we first examined the ability of differentially activated DCs injected in the footpad to migrate to draining LNs. Carboxyfluorescein succinimidyl ester (CFSE)-labeled DCs were left unstimulated or stimulated with either LPS or HDM, and then injected subcutaneously into the footpads of mice. The size of the draining LNs increased following injection of DCs compared to the phosphate-buffered saline (PBS)-injected control mice, but activated DCs migrated to a greater extent to the LN than unstimulated DCs (Fig. 6e). Notably, LPS and HDM promoted DC migration to a similar extent (Fig. 6e, right), indicating that migratory ability was not entirely dependent on a pro-inflammatory phenotype. HDM-activated DCs treated with 2-DG prior to injection displayed reduced migration to the draining LN (Fig. 6f), establishing a requirement for glycolysis for effective DC migration in vivo. We confirmed this decrease in migratory ability after 2-DG treatment was not due to reduced uptake of HDM by the DCs (Supplementary Fig. 6).

Finally, to examine the migration of endogenous DCs in vivo, we administered HDM intranasally and monitored DC migration from the lung to the mediastinal LN^23^. At the time of stimulation, animals were treated with or without intraperitoneal administration of 2-DG^24^. Administration of 2-DG during allergic inflammation induced by HDM did not significantly impair the migration and accumulation of total immune cells (Fig. 6g). However, consistent with our adoptive transfer experiment (Fig. 6f), 2-DG administration reduced the migration of endogenous CD11c^+^MHCII^hi^ DCs to the lung in response to HDM (Fig. 6g). Together these data indicate that induction of glycolysis in DCs in vivo is essential to support DC motility and CCR7 oligomerization necessary for DC migration, regardless of PRR stimulus and activation phenotype.

## Discussion

Reprogramming of cellular metabolism following immune cell activation is integral to support their activation and function. The majority of studies examining cellular metabolism in DCs use TLR agonists that induce strongly pro-inflammatory activation states^7, 14, 15, 25^, which collapses mitochondrial respiration and promotes aerobic glycolysis. By examining DCs stimulated with differential TLR agonists, we demonstrate that the bioenergetic profile of DCs is far more heterogeneous than previously reported. While strong pro-inflammatory activators, such as LPS, Zym, and curdlan, collapse ATP-coupled mitochondrial respiration and promote a prominent switch to glycolysis, DCs activated with TLR agonists that promote alternative DC activation with low inflammatory potential retain mitochondrial respiration. Using ^13^C-glucose-tracing techniques, we demonstrate differential use of glucose-derived carbon in DCs stimulated with differential DC agonists, indicating diverse metabolic pathways usage by DCs depending on activation stimulus. The common metabolic link between highly and weakly inflammatory DCs is the immediate induction of glycolysis. We demonstrate that glycolysis is required for DCs to efficiently migrate in vitro, to retain their characteristic dendritic morphology, and to home to secondary lymphoid organs in vivo. Mechanistically, CCR7 oligomerization, which is essential for efficient DC migration^22^, was found to be highly dependent on glycolysis. Together our data implicate the early upregulation of glycolysis as a key mechanism supporting DC migration, in addition to supporting a pro-inflammatory phenotype of strongly activated DCs.

Previous work has established that inflammatory signals promote a metabolic shift to glycolysis in DCs^7, 14^. Our data here indicate that induction of glycolysis is not limited to DCs that acquire a highly pro-inflammatory phenotype. DCs treated with HDM, which induce Th2 differentiation^23^, did not exhibit a detectable inflammatory phenotype, but strongly induced the activation of TBK, AKT, mTORC1, and mTORC2, and showed an early increase in glycolysis (Figs. 2 and 4c)^14^. In contrast to LPS stimulation, HDM-activated DCs maintained OXPHOS and SRC similar to unstimulated DCs. SRC is linked to the total mitochondrial capacity available to cells, and can be used under conditions of stress to produce energy and maintain cell viability^26^. The lack of SRC in LPS-stimulated DCs limits their adaptability when glucose levels are limiting^15^. Thus, while early glycolysis stimulated by all TLR agonists supports DC migration, the greater SRC in alternatively activated DCs argues that they can engage mitochondrial respiration for ATP production and are more metabolically flexible than pro-inflammatory DCs. In fact, in many experiments, Zym- and curdlan-activated DCs retained some coupled respiration while increasing glycolysis (Fig. 1c) suggesting that complete loss of OXPHOS may be unique to inflammatory DCs stimulated with certain agonists such as LPS. Of note, results obtained using ZymD were highly variable depending on the batch of ZymD. When ZymD was weakly activating, DCs acquired inflammatory and metabolic phenotypes similar to HDM-activated DCs; however, more stimulatory batches of ZymD sometimes led to a stronger pro-inflammatory phenotype with metabolic characteristics more similar to Zym-activated DCs.

Despite the fact that HDM induces no appreciable pro-inflammatory phenotype in DCs (as measured by upregulation of MHC, co-stimulatory molecules, and cytokine production), HDM stimulated the activation of TBK, AKT, and mTORC1/2. Whether these pathways regulate cell shape and motility in DCs beyond their involvement in glycolysis will be the subject of future study. Despite the activation of mTORC1 in HDM-activated DCs, we did not observe significant stabilization of HIF-1α or induction of iNOS. Interestingly, TBK phosphorylation levels were maintained in HDM-stimulated cells even after AKT and mTORC1/2 activity had subsided suggesting that TBK may have another role in HDM-activated DCs at later time points.

We confirm that long-term metabolic reprogramming is modulated in part by HIF-1α in pro-inflammatory DCs^21, 27^. HIF-1α is stabilized under conditions of hypoxia and promotes expression of glycolytic genes to allow cells to generate more energy by glycolysis in the absence of oxygen. In several immune cell types, including DCs, HIF-1α can be stabilized by LPS stimulation even under normoxic conditions, consequently promoting aerobic glycolysis^28–30^. Jantsch et al.^25^ demonstrated that HIF-1α regulates DC activation by LPS, and our results here demonstrate that HIF-1α protein also accumulates when DCs are stimulated by other strong activators like LPS, Zym, and curdlan. These results are in line with a recent report demonstrating that HIF-1α is responsible for sustained glycolytic reprogramming in DCs^21^. Weakly activated DCs, such as those stimulated by HDM or ZymD, did not significantly increase HIF-1α protein expression, in agreement with the lack of glycolytic reprogramming we observed in these DCs.

The induction of NOS triggered by TLR agonists and subsequent NO production is known to inhibit mitochondrial respiration^15^, and NO promotes HIF-1α stabilization under normoxic conditions^21, 31^. We show here that the expression of iNOS and production of NO induced by a variety of DC activators, including those that engage CLRs, is directly correlated with the loss of coupled respiration. Pro-inflammatory DCs reduced coupled respiration in a dose-dependent manner, while DCs stimulated by weak activators HDM and ZymD did not appreciably increase iNOS or NO levels even at higher doses, thus maintaining coupled respiration. Therefore, we hypothesize that NO simultaneously inhibits mitochondrial function and stabilizes HIF-1α, reducing mitochondrial metabolism and promoting glycolysis, respectively. The differential usage of these metabolic pathways in DCs was dependent on the strength of activation, which was based on the nature and dose of the particular stimuli. Long-term metabolic programming at the transcriptional level stimulated by strong agonists likely depends on the presence of NO. DCs that do not express iNOS have been shown to similarly alter their metabolic activity in the presence of exogenous NO, which can be derived from other cells such as inflammatory macrophages^17, 21^, indicating that environmental factors beyond TLR agonists can help shape DC metabolism.

Here we show that glycolysis is important for the elongated shape of DCs and their ability to migrate. Migration is a crucial aspect of DC function; DCs that encounter foreign antigen must migrate to the draining LN to alert the adaptive immune system regardless of the strength or type of activation. One of our key observations is that mitochondrial bioenergetics is not sufficient to support these cellular features, but rather early activation of glycolysis is essential. Following activation, DCs upregulate CCR7, the receptor for CCL19 and CCL21, which enables them to be guided to the LNs by CCL21 produced by lymphatic endothelial cells. Recently, CCR7 oligomerization has been shown to be essential for efficient DC migration by forming a signaling hub to optimize signal transduction^22^. We found that CCR7 oligomerization was specifically dependent on glucose availability in DCs. Accordingly, blocking glycolysis was sufficient to disrupt optimal DC migration to the draining LN. However, this defect in migration is not solely due to CCR7 oligomerization but also due to a defect in motility. In vitro, we observed that DCs possess a rounded morphology in the absence of glucose, while adopting a more dynamic and elongated phenotype when glucose is present, implying strongly that glycolysis may play a crucial role in cytoskeletal remodeling.

In human prostate and breast cancer cell lines, cell migration is exploited to facilitate metastasis, and increased glycolytic activity has been shown to be associated with greater cytoskeletal rearrangements and therefore cell motility^32^. Interestingly, the ATP generated from OXPHOS was not sufficient to support cell shape and migration. A possible explanation for this requirement for glucose-derived ATP is that local ATP is used for actin polymerization at specific areas of the cytoskeleton such as for the formation of actin-rich structures lamellipodia, filopodia, and podosomes. This compartmentalization of glycolytic activity allows for rapid ATP production locally at the cytoskeleton rather than throughout the cell for the energetically demanding remodeling processes^33^. Vessel branching via migrating endothelial cells has been shown to be regulated by glycolysis, with glycolytic activator PFKFB3 playing a major role^34^. PFKFB3 was found to be enriched in lamellipodia, promoting local glycolytic activity to generate ATP at these actin-rich structures^34^. In addition to bioenergetic requirements, another mechanism for glycolytic control of cytoskeletal remodeling involves the association of the glycolytic enzyme aldolase to the cytoskeleton. A recent study describes the regulation of glycolysis by phosphoinositide 3-kinase, which activates the GTPase RAC to mobilize the cytoskeleton, and consequently releasing the actin-bound aldolase^35^. Our data show that without glycolysis, there is little motility of the cytoskeleton, suggesting a feed-forward loop may exist where glycolytic activity is needed for cytoskeletal reorganization necessary to release aldolase to further increase glycolysis. Additional work will be necessary to reveal how glycolysis can support the cytostructure and motility of DCs.

Inhibiting glucose availability may also alter signaling pathways in DCs that affect their migratory responses. AMPK, a cellular energy sensor that is activated in response to increased AMP:ATP or ADP:ATP ratios, can antagonize mTORC1 activity^36^. mTORC1 inhibition can affect cytoskeletal dynamics by preventing the protein expression of small GTPases^37^. mTORC2 also regulates cytoskeletal reorganization via protein kinase Cζ activity^38^. Therefore, altered AMPK and/or mTOR activity could contribute to the observed defects in cell shape, motility, and migration following glucose deprivation or 2-DG treatment. Additionally, since in vivo administration of 2-DG can affect multiple cell types, it is possible that defects in other cells are contributing to the migratory defect observed in these experiments.

Defined metabolic pathways are increasingly being shown to direct specific cellular functions. Previously, induction of glycolysis was thought to primarily support the bioenergetic and catabolic demands of a highly pro-inflammatory phenotype^14^. We demonstrate that regardless of activation phenotype, DCs increase glycolysis upon activation, and that glycolysis, and not mitochondrial bioenergetics, is essential to maintain DC cell shape, promote oligomerization of the chemokine receptor CCR7, and enable DC migration. Our data suggest that nutrient competition in chronic inflammatory and in particular, tumor microenvironments, in addition to inhibiting DC activation, may also interfere with DC migratory capacity, limiting their ability to migrate to draining LNs to orchestrate adaptive immune responses.

## Methods

### Mice

Female wild-type C57BL/6 mice or BALB/C were purchased from Charles Rivers Laboratories at 6–8 weeks of age (Montreal, QC, Canada). Animals were maintained in a specific pathogen-free environment. All experiments were conducted following the guidelines of the Canadian Council of Animal Care, as approved by the McGill University Animal Care Committee. Animals were randomly assigned to the different treatment groups. Investigators were not blinded.

### In vitro BMDC generation and stimulation

Bone marrow extracted from C57BL/6 mice was cultured in the presence of 20 ng/mL granulocyte-macrophage colony-stimulating factor (PeproTech) in “complete DC medium” (CDCM) containing RPMI-1640 (Corning) medium with 10% fetal calf serum (FCS) (Hyclone), 2 mM l-glutamine (Wisent), 100 U/mL penicillin-streptomycin (Wisent), and 0.01% β-mercaptoethanol (Gibco). After 8–10 days, DCs were collected, seeded at 2 million cells/mL, and stimulated where indicated with LPS (O111:B4 Sigma-Aldrich), HDM (low endotoxin from Greer Laboratories), curdlan (InvivoGen), Zym (InvivoGen), or Zym-depleted^39^ (InvivoGen). HDM was labeled with Alexa Fluor 647 in indicated experiments with the Alexa Fluor 647 Protein Labeling Kit (Invitrogen). Glucose-withdrawal experiments used glucose-free RPMI-1640 (Wisent) supplemented with 10% dialyzed FCS (Wisent), 2 mM l-glutamine, 100 U/mL penicillin-streptomycin, and 0.01% β-mercaptoethanol.

### Splenic DC isolation

Flt3L-expressing B16 cells were subcutaneously injected into C57BL/6 mice (3.5 × 10^5^ cells/mouse) in order to expand the DC population^40^. After approximately 2 weeks, the tumor-bearing mice were sacrificed and the spleens were harvested for DCs. Spleens were digested with 1 mg/mL collagenase and 10 µg/mL DNase I (Roche), and red blood cells lysed with ACK lysis buffer. Splenic DCs were purified using the Pan DC Isolation Kit (Miltenyi), according to the manufacturer’s protocol.

### Seahorse assay

An XF-96 Extracellular Flux Analyzer (Seahorse Bioscience) was used to analyze real-time changes of the ECAR and OCR. DCs were seeded in XF-96 cell culture plates at 7.5 × 10^4^ cells/well in CDCM with or without stimuli. After incubation for the indicated time points, the media was removed and replaced with warmed unbuffered “Seahorse medium” (XF Assay Base Medium with 10 mM glucose, unless specified glucose-free, 10% FCS, and 2 mM l-glutamine) at pH 7.4. Where indicated, the DCs were treated with 1 μM oligomycin, 1.5 μM FCCP, and 100 nM rotenone with 1 μM antimycin A (all Sigma-Aldrich). For analyzing real-time changes in ECAR and OCR immediately upon activation, DCs were plated in XF-96 cell culture plates at 7.5 × 10^4^ cells/well in “Seahorse medium”, and the stimuli were injected into the wells by the XF analyzer.

### Metabolite analysis

Nova Bioanalyzer: Supernatant was collected from BMDCs stimulated for 18 h, and spun down to remove any cells and debris. Supernatants were then run on the Nova Bioanalyzer to measure the levels of various metabolites, including lactate and glucose.

Gas chromatography-mass spectrometry analysis of ^13^C metabolites: Gas chromatography-mass spectrometry (GC-MS) metabolite analysis was conducted as previously described^41^. Briefly, 5 × 10^6^ BMDCs were cultured with indicated activators in CDCM for 2 h. Following activation, the medium was changed to glucose-free RPMI (with 10% dialyzed FCS, Wisent) containing 11 mM U-[^13^C]-glucose (Cambridge Isotope Laboratories) for 2 h. Metabolites were extracted from cells using dry ice-cold 80% methanol, followed by sonication and removal of cellular debris by centrifugation at 4 °C. Metabolite extracts were dried, derivatized as tert-butyldimethylsilyl esters, and analyzed via GC-MS as previously described^42^. Uniformly deuterated myristic acid (750 ng/sample) was added as an internal standard following cellular metabolites extraction, and metabolite abundance was expressed relative to the internal standard and normalized to cell number. Mass isotopomer distribution was determined using a custom algorithm developed at McGill University^43^.

Liquid chromatography-mass spectrometry analysis of nucleotides: Nucleotide levels were determined by liquid chromatography-mass spectrometry (LC-MS), as previously described^19^. BMDCs were cultured with or without glucose for 4 h, and then washed with cold 150 mM ammonium formate solution pH of 7.4 and extracted with 600 μL of 31.6% MeOH/36.3% acetonitrile (ACN) in H_2_O (v/v). Cells were lysed and homogenized by bead-beating for 2 min at 30 Hz using a 5 mm metal bead (TissueLyser II—Qiagen). Cellular extracts were partitioned into aqueous and organic layers following dimethyl chloride treatment and centrifugation. Aqueous supernatants were dried by vacuum centrifugation with sample temperature maintained at −4 °C (Labconco, Kansas City, MO, USA). Pellets were subsequently resuspended in 25 μl of H_2_O as the injection buffer. For semiquantitative targeted metabolite analysis of mono-, di-, and tri-phosphate nucleoside, samples were injected onto an Agilent 6430 Triple Quadrupole (Agilent Technologies, Santa Clara, CA, USA). Chromatography was achieved using a 1290 Infinity ultra-performance LC system (Agilent Technologies) consisting of vacuum degasser, autosampler, and a binary pump. Separation was performed on a Scherzo SM-C18 column 3 μm, 3.0 × 150 mm (Imtakt Corp, JAPAN) maintained at 10 °C. The chromatographic gradient started at 100% mobile phase A (5 mM ammonium acetate in water) with a 5 min gradient to 100% B (200 mM ammonium acetate in 20% ACN/80% water) at a flow rate of 0.4 ml/min. This was followed by a 5 min hold time at 100% mobile phase B and a subsequent re-equilibration time (6 min) before next injection. A sample volume of 5 μL of sample was injected for analysis. Sample temperature was maintained at 4 °C before injection.

The mass spectrometer was equipped with an electrospray ionization source and samples were analyzed in positive mode. Multiple reaction monitoring (MRM) transitions were optimized on standards for each metabolite quantitated. Transitions for quantifier and qualifier ions are described in Table 2 below. Gas temperature and flow were set at 350 °C and 10 l/min, respectively, nebulizer pressure was set at 40 psi and capillary voltage was set at 3500 V. Relative concentrations were determined by integrating area under the curve for the quantifying MRM transition and compared to external calibration curves. Data were analyzed using MassHunter Quant (Agilent Technologies).

**Table 2:** Agilent 6430 Triple Quadrupole MRM transitions of nucleosides

Compound	Precursor ion (*m*/*z*)	Quantifier ion (*m*/*z*)	Qualifier ion (*m*/*z*)
ATP	508.0	136.0	410.1
ADP	428.0	136.0	348.1
AMP	348.0	136.1	118.9
CTP	484.0	112.1	97.1
CDP	404.0	112.0	69.0
CMP	324.0	112.1	69.1
TTP	483.0	81.1	53.0
TDP	403.0	81.1	207.0
TMP	323.0	207.1	126.9
GTP	524.0	152.0	134.9
GDP	444.0	152.0	97.0
GMP	364.0	152.1	97.0
ITP	509.0	137.0	97.1
IDP	429.0	137.0	97.0
IMP	349.0	137.0	110.0
UTP	485.0	97.1	113.0
UDP	405.0	97.0	69.2
UMP	325.0	97.0	113.0

All LC-MS-grade solvents and salts were purchased from Fisher (Ottawa, ON, Canada): dichloromethane, water (H_2_O), ACN), methanol (MeOH) and ammonium acetate. The authentic metabolite standards were purchased from Sigma-Aldrich Co. (Oakville, ON, Canada).

### Enzyme-linked immunosorbent assay

The cytokines IL-6, IL-12p40, TNFα, and IL-10 were measured in the supernatant of activated cells as described in the Ready-Set-Go!^®^ ELISA protocol from eBioscience.

### Flow cytometry

Antibodies used for flow cytometry analysis of surface marker expression on BMDCs are the following: anti-CD11c (N418); anti-CD40 (1C10); anti-CD80 (16-10A1); anti-CD86 (GL1); and anti-I-A/I-E (M5/114.15.2) (all eBioscience). Antibodies used for flow cytometry analysis of cells from mediastinal LNs are the following: anti-CD19 (eBio1D3); anti-F4/80 (BM8) (eBioscience); anti-CD3ε (145-2C11); anti-CD45 (30-F11); anti-CD11c (N418); and anti-I-A/I-E (M5/114.15.2) (BioLegend). For staining of intracellular protein iNOS (C-11) (Santa Cruz Biotechnology), samples were first fixed with IC Fixation Buffer (eBioscience) and stained in Permeabilization Buffer (eBioscience). Samples were acquired on the BD LSR Fortessa and data analyzed using FlowJo software.

### Live imaging

DCs were seeded at 3 × 10^4^ cells/well in complete DC medium with or without 10 mM glucose in an eight-chamber cover glass plate (Lab-Tek). Cells were allowed to settle at 37 °C for 4 h, and then imaged using a ZEISS LSM700 confocal microscope and a ×20 0.8 numerical aperture objective lens. Cells were imaged every 3 min for 2 h in a humidified chamber with 5% CO_2_ and heated to 37 °C. Either 10 mM glucose or 10 mM 2-DG was added as a bolus where indicated, and imaged for another 3 h. ZEN black was used to obtain images from ZEISS LSM700 confocal microscope and movies were analyzed using ZEN blue and ImageJ/Fiji software (National Institute of Health). Shape descriptors were calculated using the Analyze Particles tool in ImageJ/Fiji and in-house custom macros. Aspect ratio was calculated as the ratio of the major axis (*d*) to the minor axis (*s*) (AR = *d*/*s*) and values are ≥1. Roundness was calculated by the equation *R* = 4*A*/(*πd*^2^), where *A* is area, and has values between 0 (oblong) and 1 (perfect circle).

### Immunoblot

Protein lysates were prepared in 1% CHAPS lysis buffer with protease inhibitor and phosphatase inhibitor, then quantified by BCA assay (Pierce). Samples were run on 10% gels by SDS-polyacrylamide gel electrophoresis, and proteins were transferred by electrophoretic wet transfer to polyvinylidene fluoride membranes. Membranes were blot for p-TBK Ser172 (D52C2), p-AKT Ser473 (D9E) and Thr308 (D25E6), total AKT (C67E7), p-S6K Thr389, p-S6 Ser240/244, β-actin (all Cell Signaling), and HIF-1α (Cayman Chemicals), all diluted to 1:1000 except for HIF-1α (1:500). Incubation with primary antibody overnight was followed by incubation with horseradish peroxidase-linked antibody to rabbit IgG for 45 min. Enhanced chemiluminescence (Perkin Elmer or Amersham) was used to develop the blots. Scans of the original blots are in Supplementary Fig. 7.

### Quantitative RT-PCR

RNA was purified from cells using TRIzol (Invitrogen) and cDNA was synthesized with a reverse transcription kit (Applied Biosystems) to perform a SYBR-based real-time PCR (Applied Biosystems) with primers (Table 3) from Integrated DNA Technologies. Data were generated using the ΔΔCq method. Relative gene expression was normalized to that of hypoxanthine-guanine phosphoribosyltransferase (HPRT).

**Table 3:** Sequences of primers for quantitative RT-PCR

Gene	Sequence
*Glut1*	Forward: CTGGACCTCAAACTTCATTGTGGGReverse: GGGTGTCTTGTCACTTTGGCTGG
*Hk2*	Forward: CCGTGGTGGACAAGATAAGAGAGAACCReverse: GGACACGTCACATTTCGGAGCCAG
*Pkm2*	Forward: GGTATCGCAGCAGGAACCGAAGTACReverse: GCTGGGTCTGAATGAAGGCAGTC
*Gapdh*	Forward: GTCGGTGTGAACGGATTTGReverse: TAGACTCCACGACATACTCAGCA
*Ldha*	Forward: TGTCTCCAGCAAAGACTACTGTReverse: GACTGTACTTGACAATGTTGGGA

### Transwell assay

DCs incubated for 4–6 h in specified conditions were seeded in the inserts of 24-well Transwell plates (5 μm polycarbonate membrane; Costar) at 4 × 10^5^ cells/insert. CCL21 (250 ng/mL) in media with or without glucose was added to the bottom chambers. Cells were allowed to migrate for 2 h at 37 °C. Cells that migrated into the bottom chambers and cells remaining in the insert were collected and counted on the BD LSR Fortessa using counting beads (123count eBeads, eBioscience). Results are expressed as the percentage of cells that migrated of total cells.

### In vivo migration experiments

Footpad injection: BMDCs were stained with CFSE and stimulated for 6 h with the indicated activators. After 6 h, cells were collected and resuspend in PBS at 20 × 10^6^ cells/mL. In all, 5 × 10^5^ (25 μL) cells were injected subcutaneously into each rear footpad of C57BL/6 mice. After 45 h, the draining popliteal LNs were isolated (popliteal LNs from each mouse were pooled) and digested with DNase I (10 μg/mL) and collagenase D (1 mg/mL). CFSE^+^ cells in the dLNs were detected by flow cytometry and cells were counted using 123count eBeads (eBioscience).

HDM-induced allergic asthma model: To investigate the effects of 2-DG on DC activation and migration, female Balb/c (7–8 weeks) were briefly anaesthetized with isoflurane and treated intranasally with 40 µg of low-endotoxin HDM (LE-HDM; Greer) in a volume of 30 µL. Mice were injected intraperitoneally with 500 mg/kg body weight 2-DG or control saline in a volume of 200 µL, daily, starting 1 day prior to LE-HDM delivery. Mice were sacrificed 72 h after application of LE-HDM, and mediastinal LNs were collected for flow cytometry analysis. CD11c^+^MHCII^hi^ cells were gated on viable CD45^+^ cells from which cells expressing CD19, CD3ε, and F4/80 were excluded.

### Fluorescence resonance energy transfer

CCR7 oligomerization was determined by FRET. BMDCs were stimulated in the presence or absence of glucose (10 mM) or 2-DG (10 mM) for 6 h. Cells were stained for 45 min with phycoerythrin (PE)-labeled CCR7 and allophycocyanin (APC)-labeled CCR7 (clone 4B12; eBioscience) at a 1:200 dilution in the presence of Fc block at 37 °C. Samples were washed and acquired on the BD LSR Fortessa without compensation. FRET was calculated as previously described^44^, using the following formula:

$$\begin{array}{l}{\mathrm{FRET}}\;{\mathrm{unit}} = \left( {E3_{{\mathrm{both}}} - E3_{{\mathrm{none}}}} \right)\\ - \left( {\left[ {E3_{{\mathrm{APC}}} - E3_{{\mathrm{none}}}} \right] \times \left[ {E2_{{\mathrm{both}}}/E2_{{\mathrm{APC}}}} \right]} \right) - \left( {\left[ {E3_{{\mathrm{PE}}} - E3_{{\mathrm{none}}}} \right] \times \left[ {E1_{{\mathrm{both}}}/E1_{{\mathrm{PE}}}} \right]} \right)\end{array}$$

where *E*1 is the fluorescence detected at 580 nm upon excitation at 561 nm, *E*2 is the fluorescence detected at 670 nm upon excitation at 640 nm, and *E*3 is the fluorescence detected at 670 nm upon excitation at 561 nm. The positive population percentage was used for *E* stained with neither PE nor APC (*E*_none_), PE only (*E*_PE_), APC only (*E*_APC_), or both PE and APC (*E*_both_).

### Statistical analysis

Data were analyzed using GraphPad Prism software (version 6). An unpaired Student’s *t*-test was performed to determine statistical significance between two samples and significance was considered to be when *p* values were < 0.05. Stars without lines indicate significance when compared to control. A one-way analysis of variance was performed to determine statistical significance between multiple groups.

### Data availability

The authors declare that the data supporting the findings of this study are available within the paper and its Supplementary Information files.

## Electronic supplementary material

Supplementary Information

Description of Additional Supplementary Files

Supplementary Movie 1

Supplementary Movie 2

## References

[1] Kawai T, Akira S (2010). The role of pattern-recognition receptors in innate immunity: update on Toll-like receptors. Nat. Immunol..

[2] Ozinsky A (2000). The repertoire for pattern recognition of pathogens by the innate immune system is defined by cooperation between Toll-like receptors. Proc. Natl Acad. Sci. USA.

[3] Gantner BN (2003). Collaborative induction of inflammatory responses by Dectin-1 and Toll-like receptor 2. J. Exp. Med..

[4] Hammad H (2010). Inflammatory dendritic cells—not basophils—are necessary and sufficient for induction of Th2 immunity to inhaled house dust mite allergen. J. Exp. Med..

[5] Norimoto A (2014). Dectin-2 promotes house dust mite–induced T helper type 2 and type 17 cell differentiation and allergic airway inflammation in mice. Am. J. Respir. Cell Mol. Biol..

[6] O’Neill LAJ, Kishton RJ, Rathmell J (2016). A guide to immunometabolism for immunologists. Nat. Rev. Immunol..

[7] Krawczyk CM (2010). Toll-like receptor–induced changes in glycolytic metabolism regulate dendritic cell activation. Blood.

[8] Haschemi A (2012). The sedoheptulose kinase CARKL directs macrophage polarization through control of glucose metabolism. Cell Metab..

[9] Pearce EL (2009). Enhancing CD8 T-cell memory by modulating fatty acid metabolism. Nature.

[10] Lampropoulou V (2016). Itaconate links inhibition of succinate dehydrogenase with macrophage metabolic remodeling and regulation of inflammation. Cell Metab..

[11] Cameron AM, Lawless SJ, Pearce EJ (2016). Metabolism and acetylation in innate immune cell function and fate. Semin. Immunol..

[12] Ip WKE (2017). Anti-inflammatory effect of IL-10 mediated by metabolic reprogramming of macrophages. Science.

[13] Warburg O (1956). On the origin of cancer cells. Science.

[14] Everts B (2014). TLR-driven early glycolytic reprogramming via the kinases TBK1-IKKε supports the anabolic demands of dendritic cell activation. Nat. Immunol..

[15] Everts B (2012). Commitment to glycolysis sustains survival of NO-producing inflammatory dendritic cells. Blood.

[16] Pantel A (2014). Direct type I IFN but not MDA5/TLR3 activation of dendritic cells is required for maturation and metabolic shift to glycolysis after poly IC stimulation. PLoS Biol..

[17] Everts B (2012). Commitment to glycolysis sustains survival of NO-producing inflammatory dendritic cells. Blood.

[18] Kelly EK, Wang L, Ivashkiv LB (2010). Calcium-activated pathways and oxidative burst mediate zymosan-induced signaling and IL-10 production in human macrophages. J. Immunol..

[19] Ma EH (2017). Serine s an essential metabolite for effector T cell expansion. Cell Metab..

[20] Alvaro MH (2009). HIF-1α modulates energy metabolism in cancer cells by inducing over-expression of specific glycolytic isoforms. Mini Rev. Med. Chem..

[21] Lawless SJ (2017). Glucose represses dendritic cell-induced T cell responses. Nat. Commun..

[22] Hauser MarkA (2016). Inflammation-induced CCR7 oligomers form scaffolds to integrate distinct signaling pathways for efficient cell migration. Immunity.

[23] Plantinga M (2013). Conventional and monocyte-derived CD11b+dendritic cells initiate and maintain T helper 2 cell-mediated immunity to house dust mite allergen. Immunity.

[24] Sukumar M (2013). Inhibiting glycolytic metabolism enhances CD8(+) T cell memory and antitumor function. J. Clin. Invest..

[25] Jantsch J (2008). Hypoxia and hypoxia-inducible factor-1α modulate lipopolysaccharide-induced dendritic cell activation and function. J. Immunol..

[26] van der Windt GJW (2012). Mitochondrial respiratory capacity is a critical regulator of CD8(+) T cell memory development. Immunity.

[27] Corcoran SE, O’Neill LAJ (2016). HIF1α and metabolic reprogramming in inflammation. J. Clin. Invest..

[28] Jantsch J (2011). Toll-like receptor activation and hypoxia use distinct signaling pathways to stabilize hypoxia-inducible factor 1α (HIF1A) and result in differential HIF1A-dependent gene expression. J. Leukoc. Biol..

[29] Cheng SC (2014). mTOR- and HIF-1α–mediated aerobic glycolysis as metabolic basis for trained immunity. Science.

[30] Tannahill GM (2013). Succinate is an inflammatory signal that induces IL-1β through HIF-1α. Nature.

[31] Metzen E (2003). Nitric oxide impairs normoxic degradation of HIF-1α by inhibition of prolyl hydroxylases. Mol. Biol. Cell.

[32] Shiraishi T (2015). Glycolysis is the primary bioenergetic pathway for cell motility and cytoskeletal remodeling in human prostate and breast cancer cells. Oncotarget.

[33] Zecchin A (2015). Metabolic pathway compartmentalization: an underappreciated opportunity?. Curr. Opin. Biotechnol..

[34] De Bock K (2013). Role of PFKFB3-driven glycolysis in vessel sprouting. Cell.

[35] Hu H (2016). Phosphoinositide 3-kinase regulates glycolysis through mobilization of aldolase from the actin cytoskeleton. Cell.

[36] Saxton RA, Sabatini DM (2017). mTOR signaling in growth, metabolism, and disease. Cell.

[37] Liu L (2010). Rapamycin inhibits cytoskeleton reorganization and cell motility by suppressing RhoA expression and activity. J. Biol. Chem..

[38] Li X, Gao T (2014). mTORC2 phosphorylates protein kinase Cζ to regulate its stability and activity. EMBO Rep..

[39] Ikeda Y (2008). Dissociation of Toll-like receptor 2-mediated innate immune response to zymosan by organic solvent-treatment without loss of Dectin-1 reactivity. Biol. Pharm. Bull..

[40] Mach N (2000). Differences in dendritic cells stimulated in vivo by tumors engineered to secrete granulocyte-macrophage colony-stimulating factor or Flt3-ligand. Cancer Res..

[41] Blagih J (2015). The energy sensor AMPK regulates T cell metabolic adaptation and effector responses in vivo. Immunity.

[42] Faubert B (2014). Loss of the tumor suppressor LKB1 promotes metabolic reprogramming of cancer cells via HIF-1α. Proc. Natl Acad. Sci. USA.

[43] McGuirk S (2013). PGC-1α supports glutamine metabolism in breast cancer. Cancer Metab..

[44] Demotte N (2008). Restoring the association of the T cell receptor with CD8 reverses anergy in human tumor-infiltrating lymphocytes. Immunity.

