# Supplementary material for: Glycolytic metabolism is essential for CCR7 oligomerization and dendritic cell migration
Source: Nat Commun. 2018 Jun 25;9:2463. doi: 10.1038/s41467-018-04804-6 (PMC6018630; doi:10.1038/s41467-018-04804-6)
Supplement: Supplementary file 1 — Supplementary Information [file 41467_2018_4804_MOESM1_ESM.pdf]

Supplementary information

Glycolytic metabolism is essential for CCR7 oligomerization and dendritic cell migration

Guak *et al*.

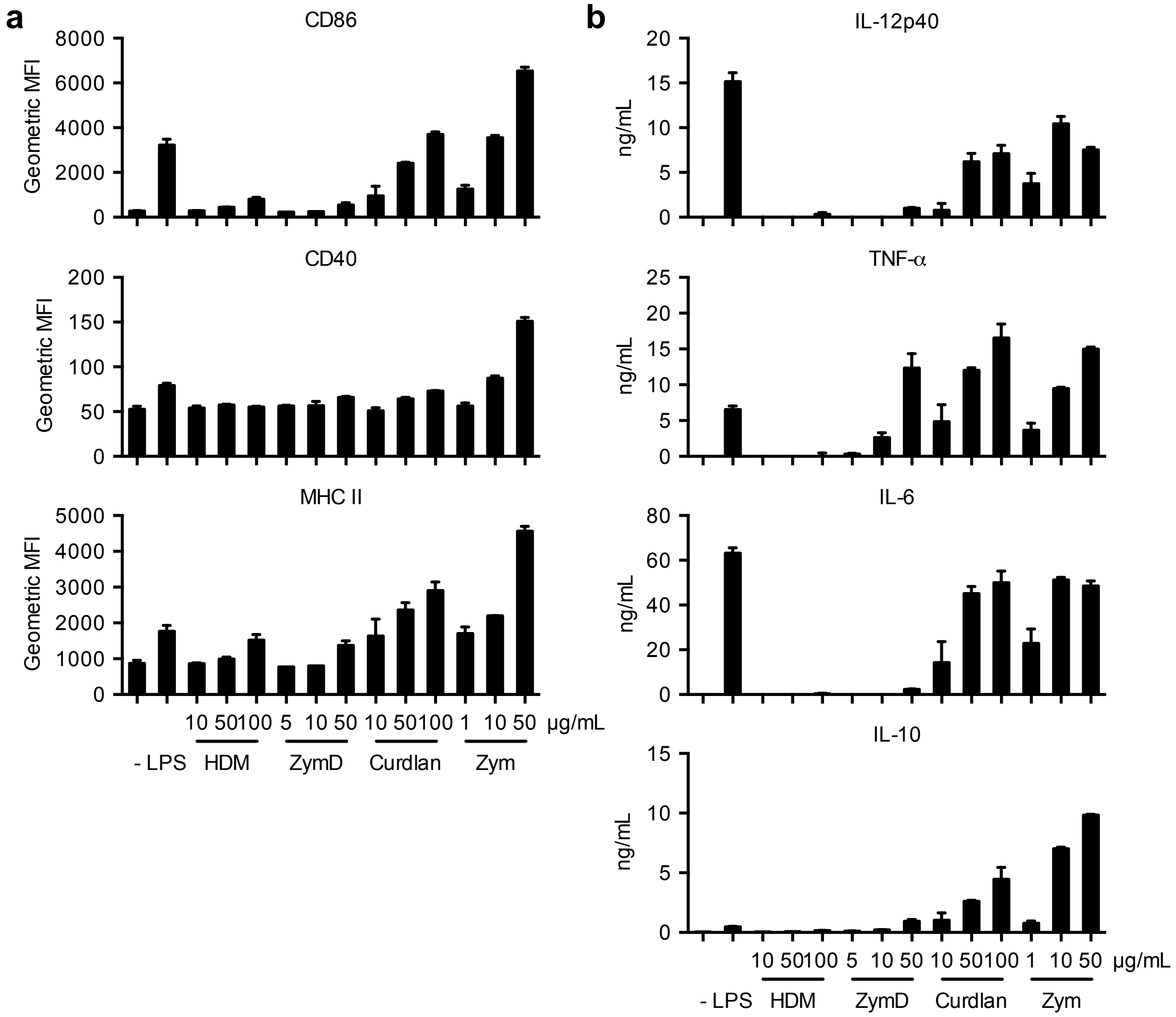

Supplementary Figure 1 – Surface marker expression and cytokine production by differentially-activated DCs.

(**a**) Expression of surface markers CD86, CD40, and MHC II and (**b**) production of cytokines IL-12p40, TNF-α, IL-6, and IL-10, were determined after stimulation with increasing doses of activators for 18h. Data in (**a,b**) are 1 experiment representative of at least 3 experiments (mean and s.d. of duplicates).

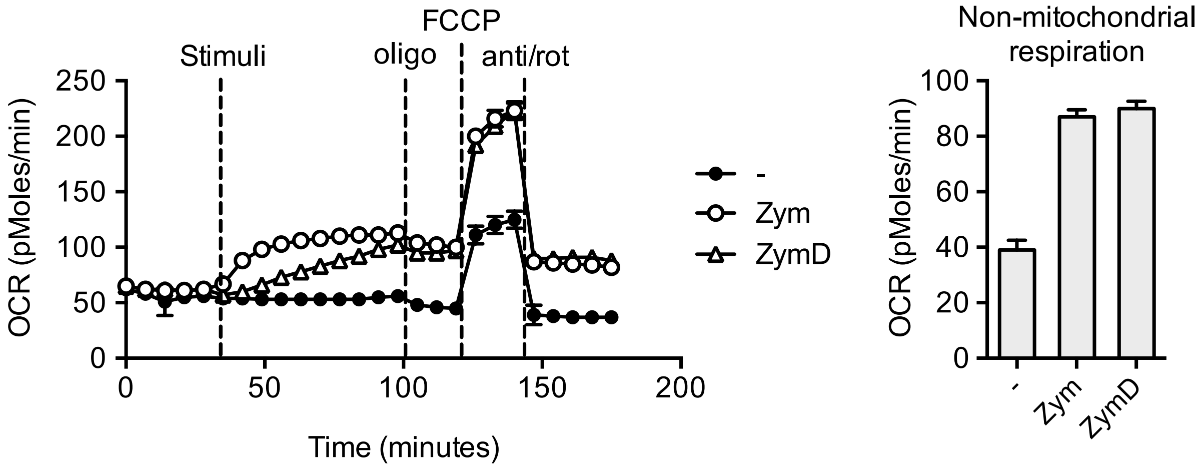

Supplementary Figure 2 – Rapid oxygen consumption induced by Zym and ZymD is primarily non-mitochondrial.

DCs were untreated or stimulated with Zym and ZymD, followed by treatment with oligomycin, FCCP, and antimycin/rotenone. The antimycin and rotenone inhibit mitochondrial respiration, and the remaining oxygen consumption is non-mitochondrial. Data shown are 1 experiment representative of 3 experiments (mean and s.e.m. of 6 replicates).

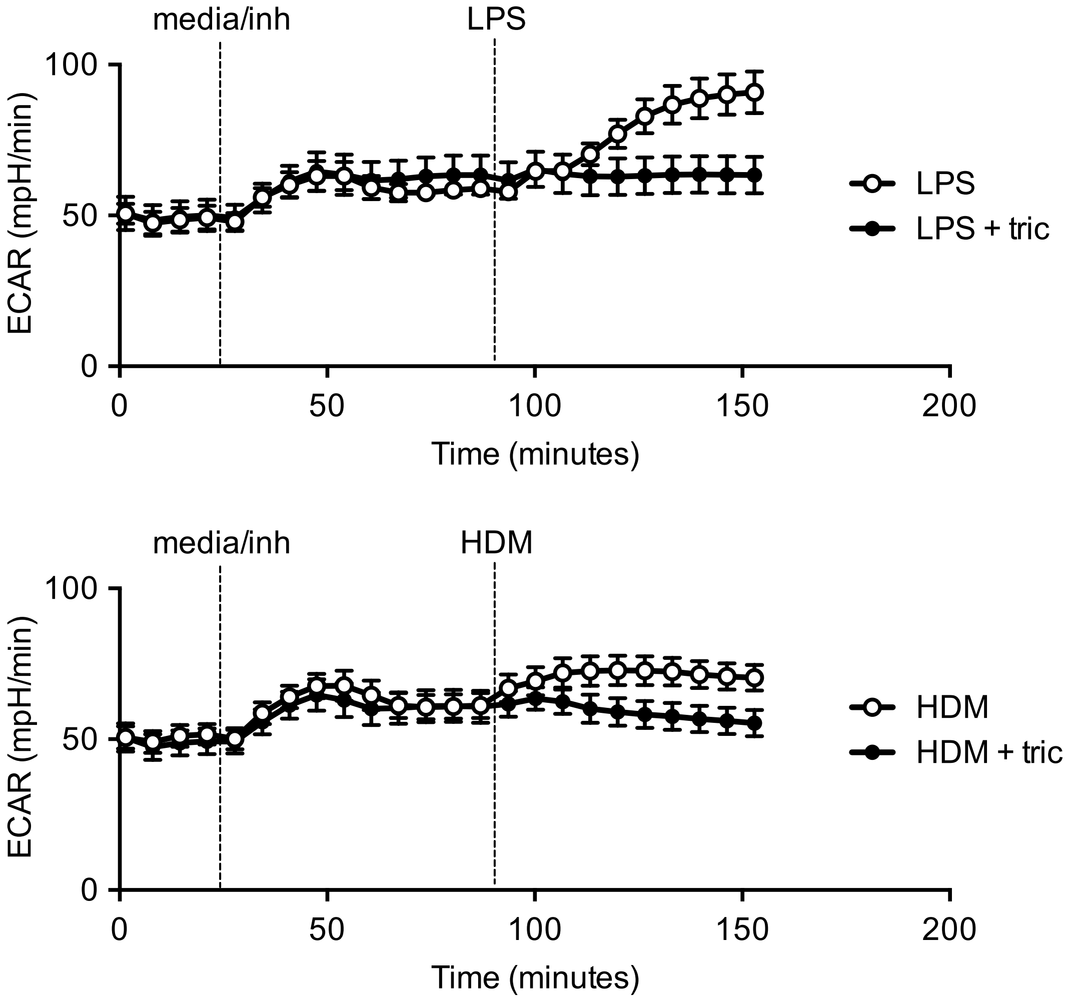

Supplementary Figure 3 – Early glycolytic flux by LPS and HDM is prevented by AKT inhibition.

ECAR of DCs treated with or without AKT inhibitor triciribine for 1h and stimulated with LPS (top) or HDM (bottom) for 1h. Data are of 1 experiment representative of 2 independent experiments (mean and s.e.m. of 6 replicates).

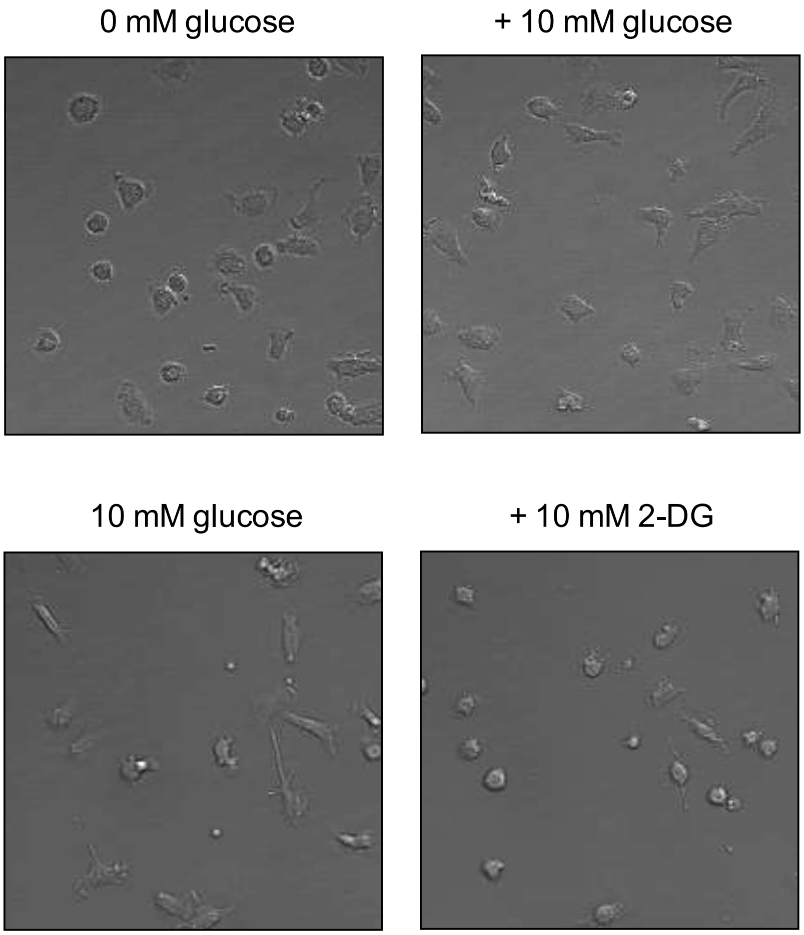

Supplementary Figure 4 – Inhibition of glycolysis results in rounded cell morphology. Representative images from the live imaging experiment analyzed in Fig. 5. DCs cultured in the absence of glucose (top), and in the presence of glucose (bottom). Glucose and 2-DG were added to the top and bottom conditions, respectfully, and cell shape was examined.

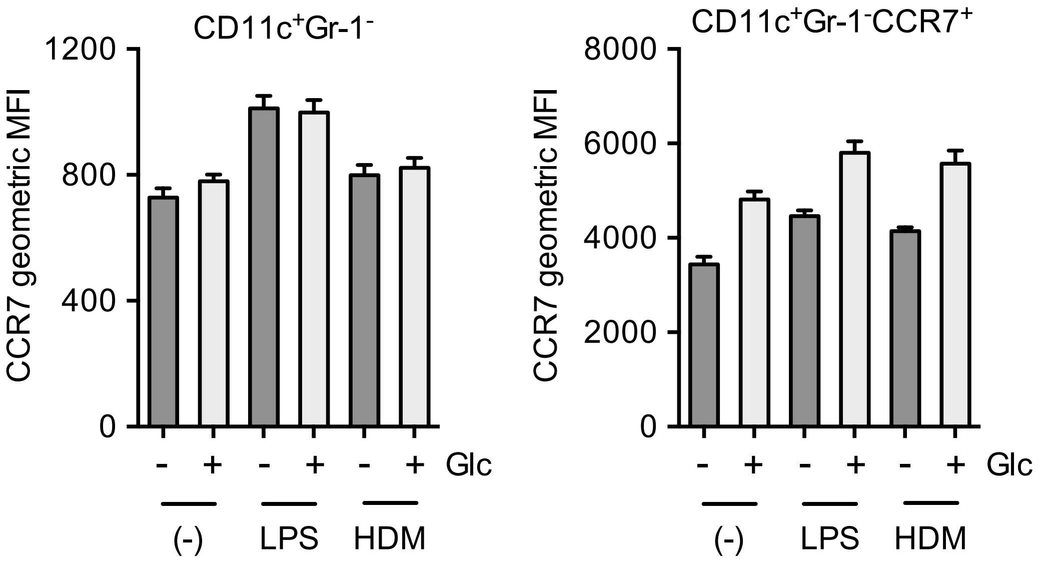

Supplementary Figure 5 – CCR7 expression on DCs.
Geometric MFI of CCR7 in the $CD11c^{+}Gr\text{-}1^{-}$ (left) and $CD11c^{+}Gr\text{-}1^{-}CCR7^{+}$ (right) populations of DCs left unstimulated or activated 6h with LPS or HDM in the presence or absence of glucose. Data are of 1 experiment representative of 3 independent experiments (mean and s.d. of triplicates).

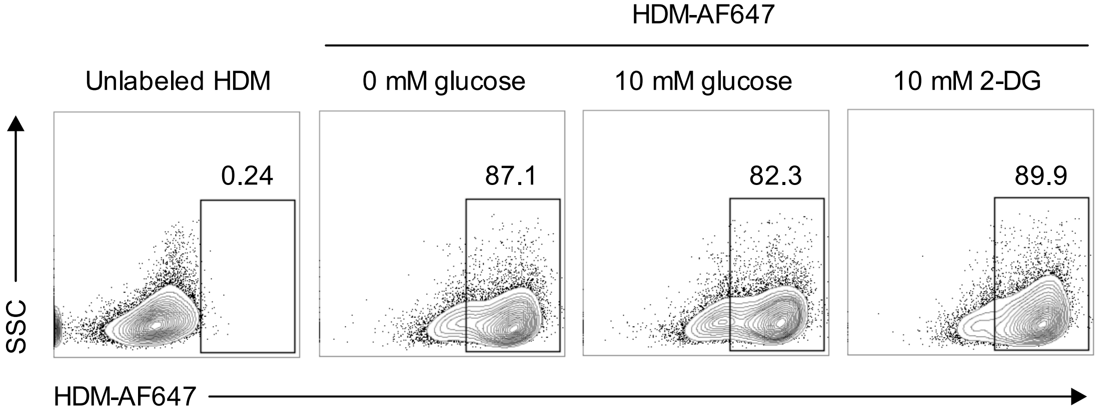

Supplementary Figure 6 – Inhibition of glycolysis does not affect phagocytosis of HDM by DCs.
HDM labeled with Alexa Fluor 647 (AF647) dye was added to DCs cultured in the presence or absence of glucose or 2-DG for 4h. Data shown are 1 experiment representative of 2 experiments.

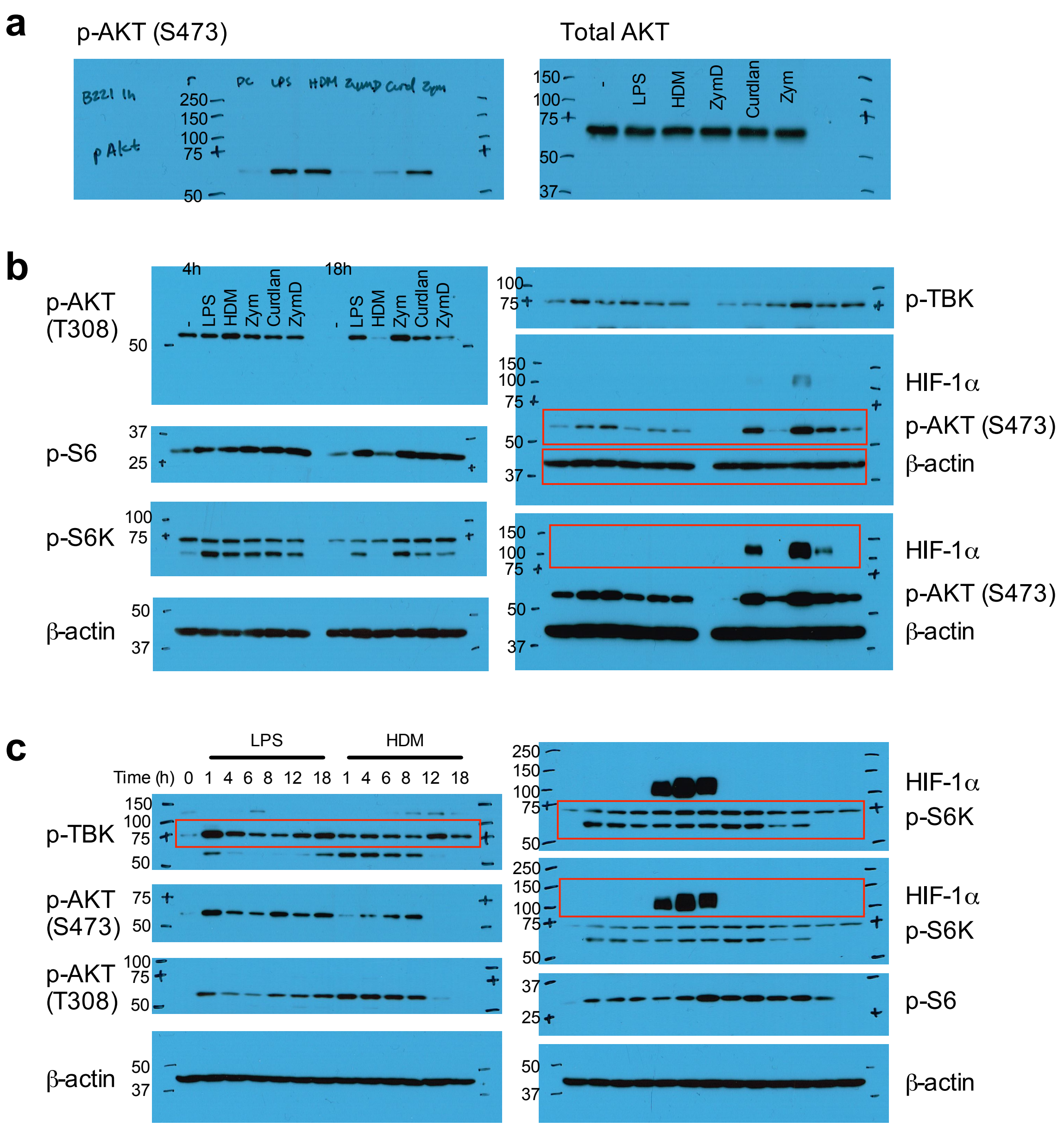

Supplementary Figure 7 – Original immunoblot scans.
Original immunoblot scans corresponding to (**a**) Fig. 2b, (**b**) Fig. 4b, and (**c**) Fig. 4c.
