# Supplementary material for: Glycolytic metabolism is essential for CCR7 oligomerization and dendritic cell migration
Source: Nat Commun. 2018 Jun 25;9:2463. doi: 10.1038/s41467-018-04804-6 (PMC6018630; doi:10.1038/s41467-018-04804-6)
Supplement: Supplementary file 2 — Description of Additional Supplementary Files [file 41467_2018_4804_MOESM2_ESM.docx]

**Description of Additional Supplementary Files**

File Name: Supplementary Movie 1

Description: DCs lacking glucose have reduced motility and more rounded morphology. Representative movie of DCs cultured in the absence of glucose for 4h. Three frames were imaged per experiment for at least 3 independent experiments.

File Name: Supplementary Movie 2

Description: DCs in the presence of glucose are motile and have elongated morphology. DCs from Supplementary Movie 1 after the addition of 10 mM glucose. Three frames were imaged per experiment for at least 3 independent experiments.
